# Antibiotic prescriptions in primary health care in a rural population in Crete, Greece

**DOI:** 10.1186/1756-0500-4-38

**Published:** 2011-02-15

**Authors:** Nikolaos Kontarakis, Ioanna G Tsiligianni, Polyvios Papadokostakis, Evangelia Giannopoulou, Loukas Tsironis, Vasilios Moustakis

**Affiliations:** 1 Agia Barbara Health Care Center, Heraklion, Crete, P.O 70003, Greece; 2 Archalohori Primary Health Care Center, Heraklion, Crete, P.O 70300, Greece; 3 University hospital of Heraklion, Heraklion, Crete, P.O 71201, Greece; 4 Technical University of Crete, Chania, Crete, P.O 73100, Greece

## Abstract

**Background:**

Antibiotic over-prescribing has generally been considered to be common in Greece, however not much is known about current antibiotic use.

**Findings:**

The aim of this study was to investigate antibiotic prescribing in a well-defined rural population of 159 adults and 99 children over a 12-month period in Crete, Greece. The daily-defined doses (DDD) for 1000 people/day (DID) were 22.1 and 24.2 for children and adults respectively. The overall DID was 23.4, markedly lower than that previously reported for Greece. The use of penicillins was 49.5% of DDD in children and 31.7% in adults. Quinolones represented 2.2% of the total antibiotics (0% in children). Prescriptions of antibiotics were more common during the 3-month period from January to March for both children and adults.

**Conclusions:**

The findings of this study confirm the seasonal distribution of antibiotics used and the predominance of prescribing for respiratory tract infections. In the area of the study, antibiotic use seems to be lower than that previously reported for Greece, probably as a result of the recently established net of well-trained primary health physicians.

## Background

Antibiotic prescribing in primary care rose steadily during the last decade world-wide, in many European countries [1-7]. Unnecessary antibiotic prescribing remains the cardinal contributing factor to the development of antibiotic resistance [4,5,7,8]. Primary health care practitioners have been shown to account for the majority of antibiotic prescribing [3]. To prevent overprescribing, detailed data on antibiotic utilization should be obtained [9-11]. The use of markers such as the daily defined doses of antibiotics (DDD), and the daily defined doses for 1000 people/day (DID) for the estimation of antibiotic use has facilitated the comparison of the findings in various countries [12]. Greece is among the European countries with the highest antibiotic prescribing and resistance to bacteria [3,4]. Information is very limited for Crete, the major island of Greece [3,13-17]. The aim of this study was to investigate antibiotic use in primary health care in a region of Crete for a period of 12 months as well as to highlight the important role of the family physicians.

## Methods

The rural municipality of Gorgolaini, prefecture of Heraklion, Crete, has a population of 3026 (1543 male, 1483 female). Primary health services consist of two public medical offices with 3 general practitioners. From an initial sample of 2394 permanent residents, a representative cohort of 330 individuals was formed by the method used for systematic samples of municipalities [18,19]. Of them, 30 (9%) denied participation, and 42 (12.7%) were not included due to lack of proper information on medical history and drug prescription. In total, 258 persons participated in the study.

The collection of data for the 12-month period from January 1 through December 31 was performed in February and March of the following year (2005). Individual health and prescription booklets were reviewed for medical visits and prescriptions, and demographic and insurance data were recorded. For each medical visit for an infection, the prescribed medication and dosage of the prescribed medication packages were recorded.

Drugs were coded according to the Anatomical Therapeutic Chemical (ATC) classification system and the doses according to the Defined Daily Dose (DDD) system [12]. The diseases were coded according to the World Health Organization-International Classification of Diseases System (WHO-ICD 10). The statistical analysis was performed using the SPSS v.17.0. Chi-square test was used in order to compare study variables. Statistical significance was accepted at the 5% level.

### Ethics

The scientific committee of the Venizeleion Hospital of Heraklion, Crete approved this study. All patients were willing to participate in the study, and were informed about the scope and the purpose of the study and gave their consent.

## Results

The age distribution of the population of the study was as follows: male, age group 0-18: 21.7%, age group 19-65: 16.2%, age group 65+: 9.6%; and female age group 0-18: 16.6%, age group 19-65: 21.3%, age group 65+ 14.6%. No significant difference was found between males and females regarding age.

The distribution of diagnoses is depicted in Table 1. Table 2 indicates the most frequently prescribed drugs, in DDDs and DIDs. The DIDs for children were 22.1 and for adults were 24.2. The use of penicillins (J01C) was 49.5% of DDDs in children and 31.7% in adults. The most frequently prescribed antimicrobial drug was amoxicillin-clavulanate (43.4% of the total DID in children). The total percentage of cephalosporins (J01DA) was 19.3% of DDDs in children and 29.7% in adults. Ceplalosporins were the most frequently prescribed antimicrobial agent in adults.

**Table 1:** Most common diagnoses in adults and children in the study

	Infection	Episodes	%
Children	Respiratory	72	82.8
	Skin-soft tissues	7	8
	Tooth abscess	5	5.7
	Gastrointestinal	2	2.3
	Others	1	1.1

Adults	Respiratory	100	71.4
	Skin-soft tissues	16	11.4
	Urinary	12	8.6
	Tooth abscess	8	5.7
	Genital	4	2.9

**Table 2:** Prescribed antibiotics as DDD and DID, and percentages

Effective Substance	Frequency	DDD	DID	% DDD
**Children**				
Penicillins J01C				
• Phenoxymethyl-penicillin J01CE02	1	6	0.17	0.8
• Amoxicillin J01CA04	5	42	1.16	5.3
• Amoxicillin-clavulanate J01CR02	28	346	9.57	43.4
Cephalosporins J01DA	24	154	4.26	19.3
Macrolides J01F	24	216	5.98	27.1
Sulphonamides QJ01EQ	1	14.4	0.4	1.8
Antifungals D01	2	10.5	0.29	1.3
Antiviral J05A	1	2.5	0.07	0.3
Antiprotozoal-anthelminthics (P01-P02)	1	6	0.17	0.8
**Total**	**87**	**797**	**22.1**	
**Adults**				
Penicillins J01C				
• Phenoxymethyl-penicillin J01CE02	2	12	0.21	0.9
• Amoxicillin J01CA04	12	162	2.79	11.5
• Amoxicilline-clavunalate J01CR02	16	272	4.68	19.3
Cephalosporins J01DA	43	418	7.2	29.7
Macrolides J01F	50	389	6.7	27.7
Quinolones J01M	5	55	0.95	3.9
Imidazoles J01XD	2	15	0.26	1.1
Sulphonamides QJ01EQ	3	30	0.52	2.1
Lincozamides J01F	2	8	0.14	0.6
Antifungals D01	5	46	0.79	3.3
**Total**	**140**	**1407**	**24.2**	

DDD = daily defined doses of antibiotics, DID = daily defined doses of antibiotics for 1000 people/day.

Macrolides represented 27.1% of the total DDD in children, and 27.7% of DDD in adults. From the group of macrolides-lincozamides (J01F) in children only clarithromycin was prescribed. In adults clarithromycin, roxithromycin, telithromycin, azithromycin and clindamycin represented 59%, 19%, 12%, 6%, and 4% respectively of the total DDD of the J01F group. In children penicillins were prescribed more often than in adults (21.4% versus 39.1%, p = 0.049), but cephalosporins and macrolides were prescribed less often compared to the adults (30.7% versus 27.6% and 35.7% versus 27.6%, respectively).

Antibiotic prescription according to infections is depicted in Table 3. In respiratory diseases penicillins (J01C) were prescribed in 48.9% and 33% of DDDs in children and adults, respectively. In children penicillins were prescribed for respiratory diseases in 48.9% of DDD and was the most commonly prescribed agent, while cephalosporins were third in rank (20.1%). In adults amoxicillin-clavulanate accounted for 20.7% and cephalosporins for 30%. Macrolides for respiratory infections were commonly prescribed (29% and 37% for children and adults, respectively. Quinolones were only prescribed in 2.2% of the total antibiotics, but were the most common agent in adult urinary tract infections (41.4% of the total DDD). Chi-square test showed no differences in (DID) and number of visits between male and female as well as between adults and children (ρ>0,05). In both children and adults, antibiotics were more commonly prescribed during January-February-March (39.6% and 32.2% of the total year, for children and adults respectively), followed by the last quarter (24.2% and 26.3% respectively).

**Table 3:** DDDs, DIDs and percentages of DDD of antibiotics according to infection

Infection	Antimicrobial agents	% children	% adults	DDD children (%)	DDD adults (%)	DID children	DID adults
**Respiratory**	Penicillins J01C	37.5%	22%	344 (48.9%)	337.5 (33%)	9.53	9.67
	Cephalosporins J01DA	29.2%	30%	142 (20.1%)	306 (30%)	3.92	5.27
	Macrolides, lincozamides J01F	31.9%	48%	204 (29%)	378 (37%)	5.65	6.51
	Sulphonamides J01E	1.4%	0	14.4 (2%)	0	0.4	0
**Urinary**	Penicillins J01C		8.3%		24 (18%)		0.41
	Cephalosporins J01DA		25%		24 (18%)		0.41
	Quinolones J01M		41.7%		55 (41.4%)		0.95
	Sulphonamides J01		25%		30 (22.6%)		0.52
**Skin-soft tissues**	Penicillins J01C	42.8%	6.3%	15.4 (52.4%)	12 (8.3%)	0.58	0.21
	Cephalosporins J01DA	28.6%	62.5%	6.5 (19.5%)	88 (60%)	0.18	1.52
	Macrolides, lincozamides J01F		12.5%		11 (7.5%)		0.19
	Other	28.6%	18.7%	7.5 (25.5%)	35.5 (24.2%)	0.21	1.09
**Genital**	Other		100%		25.5 (100%)		0.57
**Gastrointestinal**	Macrolides, lincozamides J01F	50%		12 (66.7%)		0.33	
	Other	50%		6 (33.3%)		0.17	
**Tooth abscess**	Penicillins J01C	80%	75%	30 (83.3%)	72 (90%)	0.83	1.86
	Cephalosporins J01DA	20%		6 (16.7%)		0.17	
	Macrolides, lincozamides J01F		25%		8.0 (10%)		0.14
**Various**	Other	100%		2.5 (100%)		0.07	

## Discussion

In the present study DIDs were lower than that previously recorded for Greece [3]. This could be explained in part by the fact that in the previous decade family practice in rural areas has been provided by uncertified physicians. Further in urban areas there isn't an established net of well-trained primary health physicians. Over the last decade family practice has reached a revolution and general practitioners provide primary health care services in rural areas as in our study. Our findings further confirmed the seasonal fluctuation of prescriptions, the increased use of broad spectrum antibiotics and the predominance of prescriptions for respiratory tract infections (82.8% and the 71.4% in children and adults, respectively). This is in accordance with Goosens et al study that included 32 countries [3]. In adult respiratory infections, penicillins, cephalosporins and macrolides-lincozamides shared almost equal amounts of the total DDDs. In contrast, in children penicillins were more commonly prescribed. Macrolides-lincozamides accounted for 48% for respiratory infections in adults and 31.9% in children, considerably higher than the percentages (18%) reported previously for Greece [20].

The overall DID rate was 23.4. This DID is one of the highest among European Countries, but it should be stressed that it is lower than the proportions given by the ESAC Project Group for Greece [3]. Recently, rural areas in Greece are increasingly being served by physicians specialized in general practice and this may have contributed to more rational antibiotics prescribing.

The assumption although of DDD and DID in children has several limitation problems as DDDs are normally assigned based on use in adults. The Guidelines for ATC classification and DDD assignment [21] state that the medication dose recommendations will be different depending on the age and body weight of the patient. Because of this, the WHO International Working Group for Drug Statistics Methodology has determined that it is impossible to assign pediatric DDDs, and studies that attempt to investigate drug utilization in children cannot use them. They further recommend that the general DDD be used as a measuring instrument for overall comparisons when the pediatric subgroup is difficult to identify. Therefore in our study the expression of the prescribing DDD in children might have as a result a misserpretation as it relates to an average adult weight. It can be concluded that medication use is considered greater than that estimated, as we cannot estimate precisely DDD and DID in children (DDD is the assumed average maintenance dose per day for a drug in adults). As our results showed no significant differences between adults and children it could be estimated that in children either there was a higher morbidity or there was an overuse of antibiotics. It probably means that much bigger fractions of children compared to adults were treated with antibiotics. Even although with these limitations on DDD and DID use, an overall estimation of antibiotic prescribing can be concluded.

The use of penicillins in children was satisfactory (49.5% of DIDs) but not in adults (31.7%). The overall use of penicillins in our study (38.1 of DDD) was higher than what was previously reported for Greece (31.1% and 21%) by Tzimis et al [14] and Molstad et al [4] respectively. In accordance with previous studies [3-5], the use of phenoxymethylpenicillin accounted for 1.5% of the total DDD of penicillins in children and 2.7% in adults. The increased use of wide-spectrum penicillins and particularly of amoxicillin-clavulanate has been also observed in the adjacent rural Anogia Health Centre area [13]. The predominance of broad spectrum agents has been noted in many countries and has been attributed to marketing campaigns, making physicians less sensitive to cost and quality of the prescribed drugs, and the belief that respiratory infections are an indication for antimicrobials [22-24]. All prescribed cephalosporins were of the second generation. No first-generation cephalosporins were prescribed, again a finding suggesting the unjustified overuse of broad-spectrum agents even in simple infections. No third generation cephalosporins were prescribed, mainly due to the stringent instructions for cephalosporin use that has been imposed by the Greek ministry of health since 1997. The observed increase in the use of macrolides may be explained by the availability of newer agents. Macrolides accounted for 32.6% of the total prescriptions, a percentage that was higher in comparison with other studies such as Molstad et al and Tzimis et al studies (21.9% of the total prescriptions and 22.5% of DDDs, respectively) [4,14]. Macrolides were also used very frequently in the Anogeia Health Centre area, a neighboring county of Crete [13].

The previous prescribed overuse of second generation cephalosporines and macrolides is a well known problem worldwide that leads to a Streptococcus pneumoniae resistance resulting in serious not easily treated infections [25]. Use of non recommended, more expensive, broader-spectrum antibiotics is frequent also in other studies [26]. There is growing concern that using second generation cephalosporins and macrolides may lead common pathogens to develop antibiotic resistance to Streptococcus pneumoniae, Steptococcus pyogenes and Haemophilus influenzae [3,25,27]. Further antimicrobial resistance of S.pneumoniae to penicillin at a country level is often due to macrolides and beta-lactam antibiotics [28]. A systematic review and meta-analysis on the effect of antibiotic prescribing in primary care on antimicrobial resistance showed that individuals that were prescribed an antibiotic in primary care for a respiratory or urinary infection developed bacterial resistance to that antibiotic [29]. This increases the bacterial resistance to first line antibiotics, and has as a result an increased use of second line antibiotics in the community [29].

Although quinolones can be prescribed in Greece only under specific indications, their use was not rare in our study (2.2% of the total antibiotics), mainly for adult urinary tract infections, where quinolones held first position (42%). Interestingly not a single prescription of quinolones was documented in children. Comparing our findings with those previously reported for Greece [20], a fall in the percentage of sulfonamides and an increase of quinolones was observed.

In our study there was no registration of antiprotozoal medications and only one registration of a vermifuge medicine. This finding, compatible with a previously report for Crete [30], reflects the optimal hygiene level of the area. Regarding the seasonal distribution of the medical visits, the majority of the antibiotics were prescribed during the first quarter of the year. In Goosens et al study [3] the seasonal distribution in 32 European countries had a mean increase ≥30% in Southeastern countries in the first and last quarter of the year, while the mean increase in North countries was less than 25%.

The decrease of DDD, the lack of third generation cephalosporins prescriptions, and the lack of quinolones prescribed in children are indications that proper medical education and restrictions of prescriptions can help to improve antibiotic utilization in countries with high antibiotic use. On the other hand, the increase of prescribed broad-spectrum agents such as second-generation cephalosporins and quinolones stress that much more must be done.

Further this study showed that the primary indication for antibiotic prescribing was respiratory infections, both in adults and children. In Gjelstad et al study in Norway the respiratory infections accounted for the 60% of general practitioners' antibiotic prescriptions [31]. Findings were similar in our study. In the Gjelstad et al study the antibiotic prescription has been shown to be influenced by various factors including type of infection, type of contact, being a general practitioner specialist, and years since medical exam [31]. In a retrospective cohort study that took place in UK, adults had higher probability of receiving an antibacterial prescription for a respiratory infection visit compared to children [32]. The antibacterial prescribing declined faster for younger patients than for adults after the implication of successful UK strategies for eliminating the antimicrobial use of medicines. Over the last several years a decline in antibiotics prescription in children has been reported in England [33] and this has been attributed to the significant reduction in GPs prescriptions. In our study a high antibiotic prescription for respiratory infections in children has been found, showing that further effort is needed to change GPs approach to childhood respiratory infections.

### Implications to the Greek health care system

Today, Greece spends almost 9.7% of the GDP (Gross Domestic Product) for health expenses and there is much of discussion about the present economical state, and the medication cost in Greece as well as in Europe. Our study findings should be of interest to health policy makers who are in an extremely difficult financial situation at this time, which should generate more attention to the general practitioner/family medicine specialty that now represents only the 2% of doctors.

### Strengths and limitations

This study has some limitations which should be reported. The study is a prevalence study so it does not give information about its change overtime. It is a small sample study so broad conclusions cannot be drawn. Large multicenter studies are needed in order to eliminate local factors that can influence the results. The study was conducted in only two rural areas of Crete, and therefore could not be representative of every Greek rural community, so the findings of this study could not be generalized for the whole Greek population. The use of over the counter medicines can not be excluded. The fact that we used a retrospective analysis of antibiotics in patients without interview may have as result an underestimation of the total antibiotic use.

Further the attempted comparison between adult and pediatric antibiotic use has several limitations as co-morbidity factors were not examined (diabetes, asthma etc) as well as a possibility of misestimating the results as DDDs are not recommended for children. On the other hand this is a real life study and it is among the few published regarding antibiotic use in primary health care in rural areas in Greece.

## Conclusions

In conclusion, although our findings were derived from a rather limited rural population, it suggests that well trained, primary health physicians rationally distribute antibiotics in the community setting. The findings of this study confirm the seasonal distribution of antibiotics use and the predominance of prescribing for respiratory tract infection. The overuse of cephalosporines and macrolides as revealed in our study represent a major issue for the public health as it could be associated with increased antibiotic resistance.

## Abbreviations

ATC: Anatomical Therapeutic Chemical classification system; DDD: daily-defined doses; DID: daily defined doses for 1000 people/day.

## Competing interests

The authors declare that they have no competing interests.

## Authors' contributions

IT and NK: collected, analyzed the data and prepared the first draft. NK,IT,PP,EG,LT,VM: have all participated in conception and design, and gave relevant comments. All authors have given their final approval of this version to be published.
